# Design and implementation of the first nationwide, web-based Chinese Renal Data System (CNRDS)

**DOI:** 10.1186/1472-6947-12-11

**Published:** 2012-02-28

**Authors:** Fengbo Xie, Dong Zhang, Jinzhao Wu, Yunfeng Zhang, Qing Yang, Xuefeng Sun, Jing Cheng, Xiangmei Chen

**Affiliations:** 1 Medical Systems Biology Research Center, Department of Biomedical Engineering, Tsinghua University School of Medicine, Haidian District, Beijing 100084, China; 2 National Engineering Research Center for Beijing Biochip Technology, 18 Life Science Parkway, Changping District, Beijing 102206, China; 3 Department of Nephrology, State Key Laboratory for Kidney Diseases, Chinese General Hospital of PLA, Fuxing Road 28, Beijing 100853, China; 4 State Key Laboratory for Biomembrane and Membrane Biotechnology, Tsinghua University, Haidian District, Beijing 100084, China

## Abstract

**Background:**

In April 2010, with an endorsement from the Ministry of Health of the People's Republic of China, the Chinese Society of Nephrology launched the first nationwide, web-based prospective renal data registration platform, the Chinese Renal Data System (CNRDS), to collect structured demographic, clinical, and laboratory data for dialysis cases, as well as to establish a kidney disease database for researchers and policy makers.

**Methods:**

The CNRDS program uses information technology to facilitate healthcare professionals to create a blood purification registry and to deliver an evidence-based care and education protocol tailored to chronic kidney disease, as well as online forum for communication between nephrologists. The online portal https://www.cnrds.net is implemented as a Java web application using an Apache Tomcat web server and a MySQL database. All data are stored in a central databank to establish a Chinese renal database for research and publication purposes.

**Results:**

Currently, over 270,000 clinical cases, including general patient information, diagnostics, therapies, medications, and laboratory tests, have been registered in CNRDS by 3,669 healthcare institutions qualified for hemodialysis therapy. At the 2011 annual blood purification forum of the Chinese Society of Nephrology, the CNRDS 2010 annual report was reviewed and accepted by the society members and government representatives.

**Conclusions:**

CNRDS is the first national, web-based application for collecting and managing electronic medical records of patients with dialysis in China. It provides both an easily accessible platform for nephrologists to store and organize their patient data and acts as a communication platform among participating doctors. Moreover, it is the largest database for treatment and patient care of end-stage renal disease (ESRD) patients in China, which will be beneficial for scientific research and epidemiological investigations aimed at improving the quality of life of such patients. Furthermore, it is a model nationwide disease registry, which could potentially be used for other diseases.

## Background

Chronic kidney disease (CKD) is a worldwide public health problem with an increasing incidence and prevalence, poor prognosis, and high treatment cost that affects people of all ages and demographic backgrounds [1]. The annual rate of dialysis in Mainland China was 79.1 per million people (pmp) in 2008 [2]. Renal replacement treatment for patients with end-stage renal disease (ESRD), including maintenance dialysis and transplantation, has created large economic burdens for patients and the government [3]. As the number of newly diagnosed CKD cases in China increases annually [4], it is essential to establish a central database with up-to-date inputs about various aspects of renal disease for individual cases. Unfortunately, there is no national registry system for tracking dialysis data throughout the country [5,6]. To create a shared database for dialysis centers and hospitals at a national level, the Chinese Society of Nephrology (CSN), with an endorsement from the Ministry of Health of the People's Republic of China, organized the establishment of the first nationwide, web-based renal registry system, the Chinese Renal Data System (CNRDS), to maintain the electronic records of laboratory tests and clinical practice for individual patient with CKD. The electronic portal https://www.cnrds.net was launched on May 1, 2010.

Disease registries, i.e., controlled lists of persons with a specified clinical condition and their associated data, are used to support public health and clinical research activities [7]. Such registries have been used in cancer research since the 1940s as tools to estimate cancer incidence and support etiological exploration by recording cases reported within a defined time or place [8]. Since then, multiple registries for many types of diseases have been used to assist the clinical research process [9-13]. Various national or regional renal registries, such as the United States Renal Data System (USRDS) [14], UK Renal Registry (UKRR) [15], Australia & New Zealand Dialysis and Transplant Registry (ANZDATA) [16], and the Hong Kong Renal Registry (HKRR) [17], have been developed to collect data on renal disease. One of the most important uses of a national renal registry is to track the provision of dialysis services in a country. There is an important responsibility for national renal registries to periodically publish data on the physiological and pathological condition of patients who are under dialysis treatment in an objective and anonymous fashion, as well as to enable the evaluation of the dialysis procedure and the prognosis of the patients. The variations in performance among dialysis providers are systematically documented in renal registries, which could be used for clinical audits to improve the quality of dialysis care [18]. The CNRDS was established for the above purposes, and it was expected to provide the information needed for public health, monitoring the safety of therapeutic products and services, and for clinical research. The goal of this paper was to describe the technical details of the design and implementation of this web-based registry application.

## Methods

### Objective and design

The objectives of the CNRDS are to:

1. Design and implement a consolidated renal disease data system that will provide necessary information to determine the disease burden attributable to kidney disease, as well as its geographic distribution and temporal trends in Mainland China;

2. Report on the incidence, prevalence, and mortality rates and trends of renal disease over time by primary diagnosis, treatment modality, and other sociodemographic variables;

3. Develop and analyze aggregated data that will be helpful for the examination of the prevention and progression of renal disease;

4. Stimulate and facilitate scientific research for renal disease; and

5. Provide an online communication platform for nephrologists.

**Figure 1:**
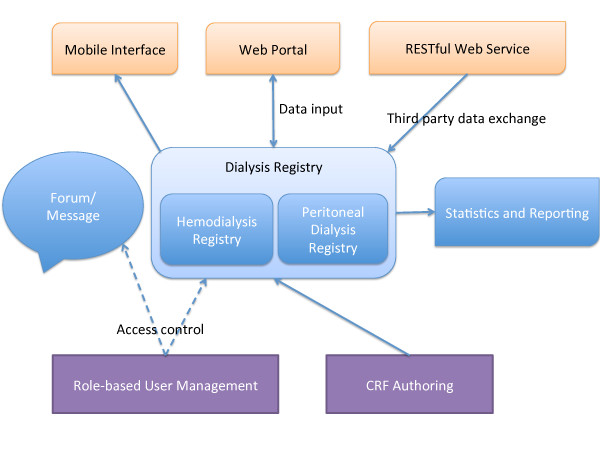
**Key components of the CNRDS**. The core dialysis registry consists of registration of both hemodialysis and peritoneal dialysis. Users interact with the system through a web-based interface; access to the content is restricted to their roles. Data in the registry can be exported for statistical analysis or generating reports

This information system allows multi-institutional users to simultaneously submit a variety of data on kidney disease in a standard, efficient, and convenient way. Figure 1 summarizes the components of the registry system, including role-based access control and user management, a case report form (CRF) authoring tool, a dialysis registry (with both hemodialysis and peritoneal dialysis registration), a statistics and reporting module, and an online discussion forum. A web-based interface is used to interact with the system. Further, a mobile interface was developed for the convenience of monitoring data through Android or iOS devices. Because several local dialysis registries have been established [5,19,20] prior to the national one, the RESTful web service can be used as a data exchange interface for automatically importing data from third party registries.

### Information architecture

The CNRDS is implemented using a JavaEE three-tier, web-based architecture: 1) a web interface that interacts with the user, 2) a middle tier that contains the application's business logic, and 3) an integration tier that consists of the enterprise resources. The CNRDS runs on an Apache Tomcat web server and has a MySQL cluster as the back-end database.

### Data collection forms

To promote the acquisition of high quality, clinically meaningful data, standard data collection forms were defined according to the blood purification standard operation procedures (SOPs) developed by the CSN [21,22], which outline CKD-related signs, symptoms, laboratory tests, and treatments that are internationally accepted to monitor onset, progression, and outcomes over the lifelong course of the disease. Nephrologists from the Registry Board of Advisors participated in the development of the CRFs in an effort to delineate the current standard of care of the patients with kidney disease and to facilitate consistent, thorough, and precise patient evaluations throughout the nephrology community. Nephrologists were given a Microsoft Excel spreadsheet for entering the required metadata information (e.g., field name, data type, value range, and presentation style) about each measurement in each case report form. An IT support team staff collected worksheets via electronic mail and then built web-based electronic forms for data collection. A hyperlink to the prototype application was given to the nephrologists along with instructions for testing and further iteration of the metadata spreadsheet. When feedback returned, the IT support team edited the electronic forms and republished them. The CNRDS utilizes CapitalBio electronic medical records (EMR) system's (CapitalBio Corporation, Beijing, China) dynamic case report form (dCRF) technology to enable modification and publishing of the CRFs on-the-fly. The case report forms created from the CNRDS are presented in Table 1. Additionally, the following administrative data elements are stored along with the CRFs: date when the case was submitted, registering institution code, and the person who submitted the data.

**Table 1:** Case report forms

Category	Case report forms	PD	HD
General	Past medical history	**✓**	**✓**
	
	Physical examination	**✓**	**✓**
	
	Vital signs	**✓**	**✓**

Diagnosis	Primary disease	**✓**	**✓**
	
	Pathology	**✓**	**✓**
	
	Complications	**✓**	**✓**
	
	Infectious Disease	**✓**	**✓**
	
	Cancer	**✓**	**✓**
	
	Anaphylaxis	**✓**	**✓**
	
	Outcome	**✓**	**✓**

Laboratory and assistant examination	Laboratory tests	**✓**	**✓**
	
	Assistant examination	**✓**	**✓**

Treatment	Erythropoietin	**✓**	**✓**
	
	Iron	**✓**	**✓**
	
	Antihypertensive drugs	**✓**	**✓**
	
	Activated vitamin D	**✓**	**✓**
	
	Calcium	**✓**	**✓**
	
	Phosphorus	**✓**	**✓**
	
	Other drugs	**✓**	**✓**

Dialysis	Peritoneal access	**✓**	
	
	Vascular access		**✓**
	
	Normalized ultrafiltration	**✓**	
	
	Dialysis prescription	**✓**	**✓**
	
	PD procedures	**✓**	
	
	Dialysis adequacy	**✓**	**✓**
	
	Peritoneal equilibration test	**✓**	
	
	Infection	**✓**	
	
	Non-infectious complications	**✓**	
	
	Blood pressure measurement		**✓**
	
	Anticoagulants		**✓**
	
	Dry weight		**✓**
	
	Combined with other dialysis therapy	**✓**	**✓**

PD indicates peritoneal dialysis, HD indicates hemodialysis, and marked means employing this CRF

The information stored in the database is fully structured. Clinical information represented in a systematically structured format is the foundation of the downstream analytical and decision support programs. Previous studies demonstrate that structured electronic medical records can result in faster and more accurate data entry, are useful in daily clinical work [23], and can be used for valid research purposes and quality assessment [24]. Our practice and other studies [25,26] have revealed that structured data entry with well-designed and intelligent user interfaces minimizes the time and effort required to capture information.

### User interface

**Figure 2:**
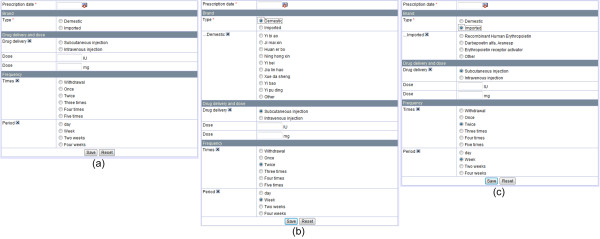
**Data collection form fragment**. The initial erythropoietin (EPO) data collection form is shown in (a). As shown in (b) and (c), the visible data elements change according to the user's selections

Web applications can support cross-platform analysis and data sharing among multiple centers of diverse technical skills and physical locations [27]. Access to shared information is possible at any time in any place with minimal infrastructure support through browser-server architecture [28]. Because healthcare professionals are accustomed to pervasive browsers and no additional training or software maintenance is required, a web-based user interface is the ideal solution for distributional data collection application. The CNRDS user interface was designed to eliminate ambiguity in the data collected and to assist users with accuracy and ease of completion. With fully structured data type definition, most elements of the collection forms are selection choices or checkboxes. Figure 2 presents a fragment of the erythropoietin (EPO) collection form as an example.

**Figure 3:**
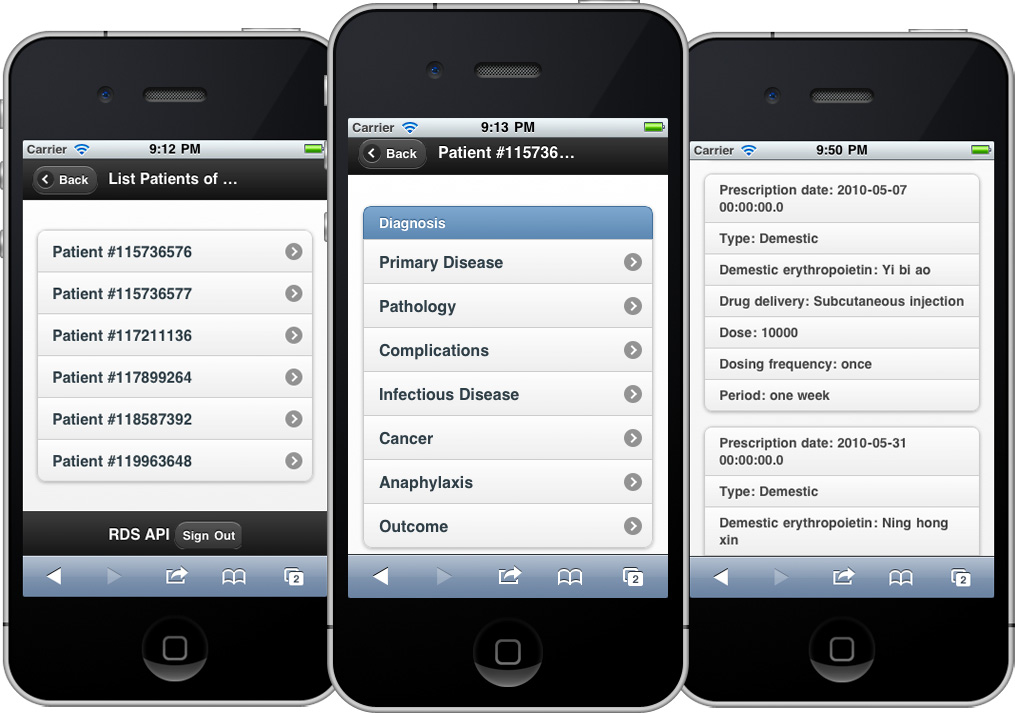
**Mobile CNRDS interface on the iPhone**. The left screenshot shows a list of patients, and the central one lists all submitted CRFs of a patient. The right screenshot displays the submitted data of a specified form

The latest generation of smartphones, such as Apple's iPhone and various Google's Android touch screen devices, are increasingly viewed as handheld computers rather than phones, due to their powerful on-board computing capability, capacious memories, large screens and open operating systems that encourage application development [29]. It is clear that the potential for mobile communication to transform healthcare and clinical intervention in the community is tremendous. Several previous studies have evaluated the use of mobile phones to support healthcare and public health interventions, notably in the collection and collation of data for healthcare research and education purposes [30,31]. Rather than developing native apps for different platforms, we built the CNRDS mobile interface using HTML5 and the jQuery mobile framework [32]. The mobile version works well on the iPhone (see Figure 3), iPad, and Android phones.

### Data validation

CNRDS is dedicated to ensuring that the data entered into the registry is accurate. Formatting and acceptable range information for each data element was provided in the spreadsheet after the experts defined the CRFs. The IT support team used this information to build logical evaluations for the data. The user's input is validated both server-side and client-side. The validation components of the web interface prevent the users from entering erroneous information into the system. A JavaScript validator checks the user's input against the validation rules before submitting the form. If a user does not enter data in a required field, the system displays error messages requesting this information prior to accepting the entry for submission to the registry. The system will not accept the data until the required fields are filled in. The same applies if the data entered is out of the expected range. For example, CNRDS recognizes the expected range for an individual's total bilirubin as between 0 and 1000 μmol/L. A warning message displays if the data entered by the user is below or above the acceptable range. This gives the user a chance to review the information he/she entered and apply any necessary corrections before submitting the data. Similar algorithms are performed server-side before the data stored in the database. Authorized batch submission from third party registries through the RESTful Web service is only validated server-side. Third party registry software providers are informed to guarantee the integrity and validity of their data.

### CNRDS security

Security issues have been addressed during the development and deployment of the application. The CNRDS system is only accessible to registered users at participating sites. To ensure that they have the authority to proceed with data entry, authorized users are issued their own unique electronic signature, i.e., a username-password combination. Each user has an appropriate level of access to the data. There are three user roles, each of which has a particular type of authority (see Table 2). All subjects' identical information collected in the CNRDS is encrypted when entered into the database, which minimizes the risk of unauthorized access to this data.

**Table 2:** CNRDS user roles and their authority

Role	Authority
System Administrator	Create/activate/suspend ministerial managers, manage institutions, and modify/publish electronic CRFs.

Ministerial manager	Create/activate/suspend provincial users, release standard documents and make announcements, and retrieve and audit all cases.

Provincial coordinator	Create/activate/suspend institutional users and retrieve and audit cases of this province.

Institutional User	Enter/update cases and retrieve and edit existing cases of his/her patients.

The system utilizes Hypertext Transfer Protocol Secure (HTTPS) for web server communication. HTTPS is a combination of the Hypertext Transfer Protocol (HTTP) with the Secure Socket Layer (SSL) protocol to provide encrypted communication and secure identification of a network web server. HTTP is unsecured and is subject to man-in-the-middle and eavesdropping attacks, which can allow attackers to gain access to website accounts and sensitive information. HTTPS is designed to withstand such attacks and is considered secure against such malfeasance [33].

We established MySQL master-slave replication for load-balance and, more importantly, for backup. Replication enables data from one MySQL database server (the master) to be replicated to one or more additional servers (the slaves). Because data is replicated to the slave(s), and the slave(s) can pause the replication process, it is possible to run backup services on the slave(s) without corrupting the corresponding master data [34]. Further, a dumped database is packaged and transferred to a backup facility located in a different network zone every night.

### Governance and technical support

The CNRDS program is governed by the CSN with support from the Ministry of Health of the People's Republic of China. The Information Technology team from the National Engineering Research Center for Beijing Biochip Technology (NERCBBT) supports all program-related technical and logistic matters encountered by the users. The IT support team also collects feedback and provides information on plans of updated versions, scheduled maintenance, bug reports, and technical tips.

## Results

**Figure 4:**
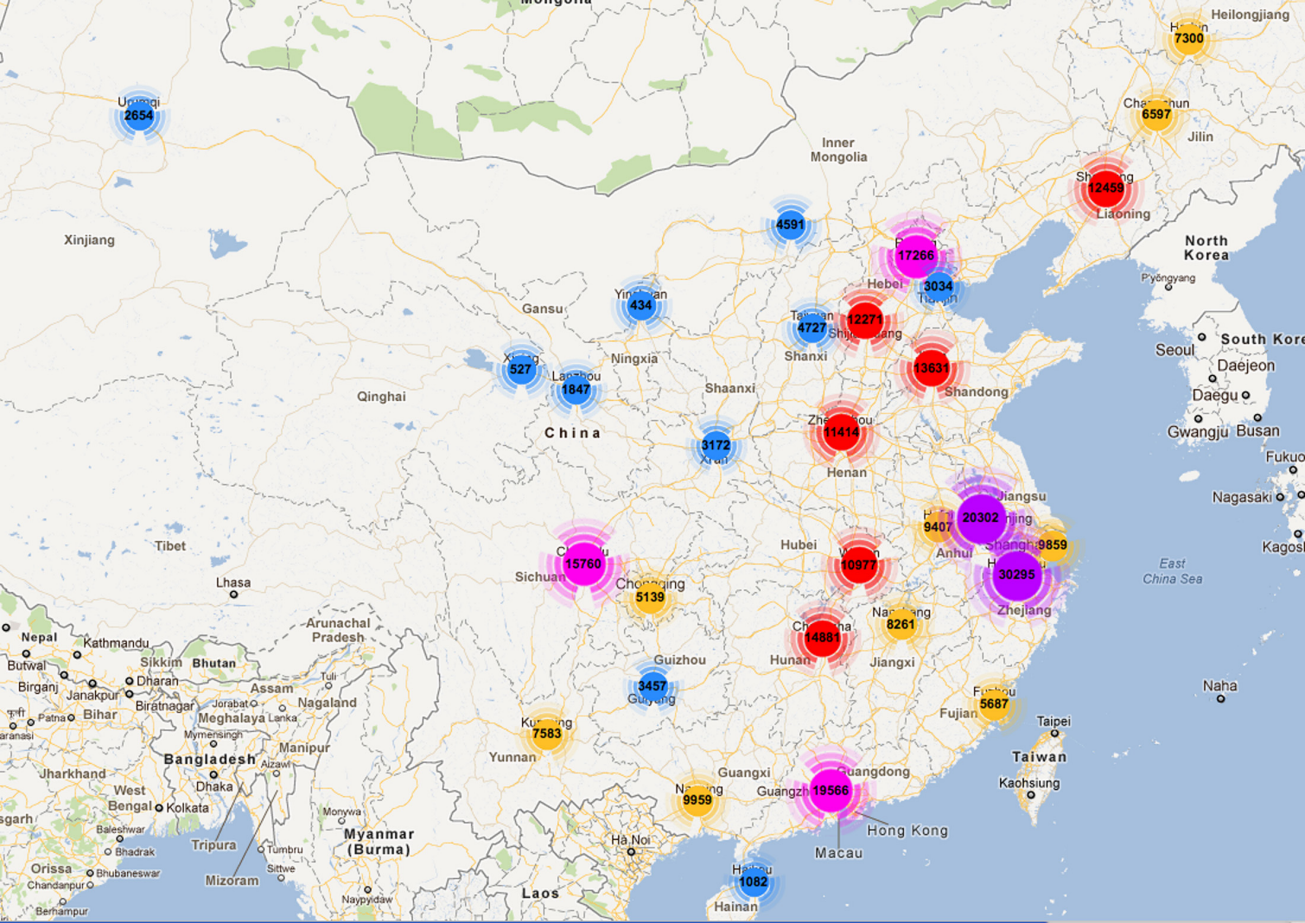
**Geographical distribution of CNRDS reported hemodialysis cases**. Up until Aug 14th, 2011, 3669 dialysis centers from 31 provinces of Mainland China have reported 274139 cases of hemodialysis. The figures in the circle represent the number of cases reported by each province

The CNRDS website has been available to the public since May 1, 2010. Prior to that, a training session was held in Beijing on April 14 for users from 31 provincial health departments and their provincial quality control centers; several hospital users also participated in the training course. To date, 3,669 dialysis centers submitted their data to the registry. Over 270,000 patients were enrolled, and this number is increasing. Of the enrolled patients, 59% were male, and the average age at initial dialysis was 51.2 years (Table 3). The geographical distribution of reported hemodialysis cases is shown as Figure 4. At the 2011 annual blood purification forum of the CSN, the CNRDS 2010 annual report was reviewed and accepted by society members and government representatives.

**Table 3:** Age and gender distributions of the enrolled hemodialysis patients

Age of initial dialysis	%
Under 30	10.06

30~39	15.00

40~49	21.48

50~59	21.40

60~69	17.79

70~79	11.75

80+	2.52

Average age, years (mean ± SD)	51.2 ± 15.8

Gender	%

Males	58.96

Females	41.04

### CNRDS features

The following key features are implemented for the current version of CNRDS: 1) collaborative access to data across multiple dialysis centers and hospitals with user authentication and role bases security, 2) intuitive electronic CRFs, 3) real-time data validation, integrity checks, and other mechanisms for ensuring data quality, 4) data audit capabilities, 5) SOP document storage and sharing, 6) central database and backups, 7) data export functions for statistical purposes, 8) data exchange interface for bulk import of data from other regional registries, and 9) online discussion forum.

### Online discussion forum

The web forum embedded within the CNRDS promotes active discussion among the renal community. People in China are familiar with online discussion forums. The professional online forum for kidney disease can facilitate interactions between nephrologists, including experts in the field. They use the platform to ask questions and share their medical experience and expertise. Feedback and suggestions for the CNRDS are also posted on the forum. The CSN uses this forum to make announcements and publish SOP documents and online surveys. From April 22nd, 2010 to Nov 15th, 2011, 2,441 threads composed of 9500 messages were exchanged on the forum. Table 4 shows a summary of topics and threads. At the first couple of months when the CNRDS just launched, physicians are not familiar with it, most of the posts are about how to use the system. After that, topics are more and more concentrated in the discussion of dialysis. Weekly digest of the forum posts are reported to the Board of the CSN. The online forum has been an important communication channel for nephrologists and had a positive influence on patient care.

**Table 4:** Summary of online forum threads*

Topics	Threads	Posts
Nephrology	1665	5390

System	503	989

Other	273	3121

Total	2441	9500

* Statistical period: April 22nd, 2010 ~ November 15th, 2011

### The CNRDS application programming interface (API)

Prior to the nationwide CNRDS, Shanghai, Beijing, and Zhejiang provinces established their own local renal registries. Indeed, these three registries have been running for several years and contain valuable data. In order to maximize the value of the previous investment, a RESTful web service API was developed for third party software providers to exchange their data with CNRDS. The RESTful web service was chosen due to its streaming capability and on-the-fly compression [35]. The data exchange format is a customized XML schema, which is downloadable as an additional file of this manuscript (Additional file 1). Each regional registry was assigned a unique API key for discrimination. Because the API is an interface between machines, third party software vendors should ensure that the data they submit is validated and complete. Data validation rules are also performed on the CNRDS server; an error code will return if the submission contains abnormal data.

## Discussion

The design and implementation of CNRDS was greatly inspired by other renal registries such as USRDS, UKRR, ANZDATA. Before establishing the national wide renal registry, the previous local registries--Shanghai Renal Registry, Beijing Renal Registry and Zhejiang Renal Registry--have been evaluated, but none of them meet the requirements of the CSN. Compare to other registries, the advantages of CNRDS are flexibility, easy to access, and social networking. A distribution of features is summarized as Table 5.

**Table 5:** Features distribution among CNRDS and other renal registries

Features	CNRDS	US RDS	UK RR	ANZDATA	SHANGHAI RR	BEIJING/ZhejiangRR*
Web based	+	+	+	+	+	

National wide	+	+	+	+		

CRF authoring tool	+					

API	+					

Mobile access	+					

Forum	+				+	

Messaging	+					

+ Possesses this feature

* Beijing and Zhejiang renal registries use the same software

### Flexibility

Depending on the nature and diversified aspects of the disease, case report forms may differ significantly among disease registries. Even within a single disease, different treatments contain distinct information, e.g., hemodialysis and peritoneal dialysis do not share the same treatment forms (see Table 1). Additionally, tests, examinations, and treatments can change. Thus, it was beneficial to develop a registry system with enough flexibility to be configured to fit different case report forms without modifying the source code. The underlying EMR system that CNRDS is based on provides such flexibility. Trained users can utilize the comprehensive online CRF authoring tool to create new case report forms, edit existing ones, and publish the modification on-the-fly without software engineers' assistance. With a generic user interfaces for data capturing and processing, the underlying database table structure and the data-processing workflow were customized specifically for hemodialysis and peritoneal dialysis. Furthermore, the system is sufficiently flexible to be configured for other disease registries. As far as we known, none of the above surveyed registries provides this kind of flexibility.

### Easy to access

The web-based user interface makes a large amount of multicenter users can easily access the CNRDS with their desktop browsers. No additional software is required to install into the client computer. When there's an update of the system, we only need to upgrade the server-side program. The end-users will get a fresh version when they login the next time. Nowadays, most renal registries are web-based. USRDS, UKRR, ANZDATA also support paper-based form submission. The Beijing and Zhejiang locale renal registries are built upon client/server architecture, which means every user who wants to submit data is required to install a specific client program.

Furthermore, the mobile interface makes it possible for physicians to access their patients' information anywhere with handheld devices.

### Social networking

Social networking is central to many Web 2.0 and Medicine 2.0 applications and involves the explicit modeling of connections between people, forming a complex network of relations, which in turn enables and facilitates collaboration and collaborative filtering processes [36]. We introduced two social networking features: online discussion and messaging. The discussion forum was opened in the purpose of getting feedbacks of the system. Lately, we found the nephrologists are willing to share their experiences with others who are doing the same work. Other renal registries barely provide this kind of features.

### Limitations

High quality data are the key to the success of a disease registry. Because there are no full-time data managers, nephrologists or nurses themselves participate in the data submission after their daily medical work. They do not have enough time to explore the system and will only submit required data. A complex system is not adequate for this type of low-frequency user. Therefore, it was important to establish a system with a simple structure and intuitive user interface. Data required by the CNRDS, such as basic patient information and laboratory tests, are partially stored in the participating institutions' hospital information systems (HISs) or clinical information systems (CISs). However, we cannot import any data from local HISs or CISs due to the variance of those information systems and hospitals' data sharing policies. Thus, it is enormously redundant to have doctors retype these data for the CNRDS.

## Conclusions

The successful implementation of the CNRDS is based on the collaborative efforts of multiple institutions with expertise in nephrology, medical oncology, pathology, epidemiology, and computer science. The CNRDS offers a number of benefits, including: 1) standardized data elements, vocabulary, and forms for data collection, 2) computerized audits and data quality control, 3) submission of data through the internet in an effective, secure, and easy to use way, and 4) the ability to exchange information with other registry systems.

The web-based CNRDS application plays a role as an online clinical information system, as well as a health administration database. Nephrologists can use the system to reorganize the process of care delivery and establish a renal registry to manage, track, and analyze large amounts of clinical information to improve decision making. The database can also potentially be used for clinical research and administrative statistics. Users can export their hospital's/province's/national data (restricted by their role) into spreadsheets for further analysis. In the near future, investigation of the data may reveal the status and trends of renal disease in Mainland China by utilizing the CNRDS system.

The establishment of the CNRDS also demonstrates that state-of-the-art information technology can facilitate implementation of a comprehensive nationwide disease registry. The underlying information system that utilizes dCRF technology is flexible and can be adapted for other disease registries at a national scale.

## Competing interests

The authors declare that they have no competing interests.

## Authors' contributions

XC and JC conceptualized and supervised this project. FX, DZ, JW, YZ, and XS designed and implemented the project. JW and QY provided technical support. FX drafted the manuscript. All authors read and approved the final manuscript.

## Authors' information

XC and JC are members of Chinese Academy of Engineering. XC is the director of Chinese PLA Institute of Nephrology, the chairman of the CSN. JC is a Cheung Kong professor of Tsinghua University School of Medicine and the director of National Engineering Research Center for Beijing Biochip Technology (NERCBBT). DZ, XS are chief physicians of the department of nephrology, Chinese PLA General Hospital. JW, QY, YZ are IT specialists at NERCBBT. FX is a Ph.D. student at Tsinghua University School of Medicine, his research interesting is focused on bringing advanced Information Technology to biomedical area to solve complex problems.

## Pre-publication history

The pre-publication history for this paper can be accessed here:

http://www.biomedcentral.com/1472-6947/12/11/prepub

## Supplementary Material

Additional file 1 **XML schema definition (XSD) file for CNRDS API**. This XSD file is the data exchange schema of the CNRDS API. Third party software vendors should submit their data in XML format with strict compliance to this schema. Click here for file
